# The Book World of Medicine and Science

**Published:** 1907-01-26

**Authors:** 


### The Book World of Medicine and Science.

A Practical Treatise on Materia Medica and Therapeutics. By John Shoemaker, M.D., LL.D. Sixth edition. (Philadelphia: F. A. Davis Company. 1906. Pp. viii. and 1,255.)

In preparing the sixth edition of his well-known volume, Dr. Shoemaker has revised the entire substance of the work and has added sections on such modern therapeutic agents as animal serums and extracts, and on *x*-rays, Finsen's light, and other mechanical methods. It may thus be consulted with confidence in reference to new as well as to older forms of treatment. The plan of the book remains essentially unchanged. It follows the custom of the majority of the manuals of materia medica rather than the newer style developed in text-books dealing in the first place with pharmacology. Hence, though it may have an old-fashioned quality, it is particularly of service to those to whom drugs are less opportunities for physiological experiment than agents to be used in the treatment of disease. We have no doubt that the sixth edition will continue the useful career which has been enjoyed by its predecessors.

Post-mortem Pathology. By Henry W. Cattell, M.D. (J. B. Lippincott Co. 18s.)

This edition is the third published only just over three years after the appearance of the first. This seems remarkable for a book dealing mainly with the technique of post-mortem work. It must, however, not infrequently happen that a medical man, called upon to perform an autopsy, is puzzled as to methods of procedure. Like so many other books published in America, this is well illustrated, and from the illustrations alone those who only examine a body after death occasionally may find the book conveys to them the greater part of the information they require. To those, however, who have more frequent opportunities of making themselves familiar with the curious aspects of morbid anatomy this book will be found a valuable guide. In making post-mortem examinations, it is scarcely necessary to remark, that care should be taken to avoid, as far as possible, leaving anything that may offend the sensitiveness of relatives. We have seen some who, when they examine the throat and tongue, carry the incision made over the chest up as far as the centre of the lower border of the jaw. Such an incision, however carefully it may afterwards be sewn up, leaves an ugly wound, which will be visible for a long distance above the shroud. An incision of this length in the neck is unnecessary. It is easy to remove the tonsils, soft palate, and tongue through an incision which does not extend higher than an inch above the level of the upper border of the sternum. Dr. Cattell evidently does not carry the incision higher than is here stated, as is shown by Fig. 90, given to illustrate the methods he adopts for the removal of the tonsils, tongue, and trachea. Illustrations are also given of what we may call the wedge-shaped method of removing the skull-cap. This method is far less likely to leave disfigurement of the face than when the brain is exposed by sawing round the skull horizontally. When the skull-cap has been removed by a horizontal incision, great care is necessary in replacing it and in sewing up the scalp to prevent the line of section of the bone showing through the skin of the forehead, because the upper portion of the skull is apt to slide over the lower. When a wedge-shaped section is made there is also the additional advantage that the skull-cap need not be sawn through, in most instances at least, below the line of the junction of the hair with the forehead. At the end of the book is a chapter upon post-mortem examinations on the lower animals.

Manual of Medicine. By Thomas Kirkpatrick Monro, M.A., M.D. (Baillière, Tindall, and Cox. 15s.)

Manuals of medicine are numerous, and it often seems mysterious where readers for them all can be. Yet we must suppose that there are readers, or they would not be published, and this manual, we notice, has reached a second edition. It is printed on good paper, and possesses a few illustrations. The descriptions of disease which it contains are clear and concise, and the method of treatment of the various subjects appears to be uniform throughout. Since the book is merely intended to present the salient features of disease and treatment, and advances no special views of the author, it calls for no detailed comment.

Aids to Medical Diagnosis. By Arthur Whiting, M.D., M.R.C.P. (London: Baillière, Tindall, and Cox. 1907. Pp. x. and 152. Fcap. 8vo. Price 2s. 6d. cloth.)

It is certainly a courageous proceeding to attempt to deal with so vast and complex a subject as medical diagnosis in a small volume of 150 pages. Yet we are bound to say that within the limits prescribed by space Dr. Whiting has succeeded wonderfully well. He possesses the art of clear and concise statement, and this, when aided, as it is here, by careful classification, makes it possible to avoid confusion even in a closely-packed paragraph. For rapid revision immediately prior to examination this book has a distinct value, and we take such a purpose to be its principal object.

School Gardening for Little Children. By Lucy L. Latter. (Swan, Sonnenschein and Co. 2s. 6d.)

Children are naturally observant, and their budding life is more or less in sympathy with all that moves around them. Yet their observations, undirected by unusual powers of concentration, rarely interest them for more than the passing moment. It is true they will often inquire the why and wherefore of what they see, but in the absence of receiving intelligent answers they soon cease to endeavour to look beyond or behind the present. Some methods of modern education have endeavoured not only to stimulate the natural pwers of observation of the child, but to use these observations as the basis of thought and reasoning. Miss Latter, the writer of this book, is one of the foremost advocates of gardens in connection with schools for young children. The book gives interesting and artistic illustrations of the children at work in their garden, and in school receiving lessons about plants. In the garden, it is needless to remark, are not only flowers and plants but insects to be seen, and these, too, are used for instruction. Neither are objects less beautiful in themselves, such as earthworms, neglected; the sun, the wind, rain and snow also, and the powers they exert, are brought in a telling manner before the children, whose minds are intelligently directed towards observing the ever-changing scenes of natural life. Miss Latter possesses not only an observant but poetic mind, and, while stimulating the mental powers of her scholars, sets also before them high ideals. A love of Nature cannot be anything but elevating. It may not transform the character, but it directs the mind into channels that must influence it—and influence it for good. A man who loves his garden is not likely to possess tastes and habits that may ruin his home.

